# Turpentine in Colic

**Published:** 1897-02

**Authors:** 


VETERINARIAN DUNLAP advises large doses of turpentine with diffusible stimulants in oil in cases of flatulent colic with threatened autointoxication. He strongly condemns morphine and atropine in these cases.—*Veterinary Record.*

